# Red Wine and Health: Approaches to Improve the Phenolic Content During Winemaking

**DOI:** 10.3389/fnut.2022.890066

**Published:** 2022-05-25

**Authors:** Ricardo Vejarano, Mariano Luján-Corro

**Affiliations:** ^1^Department of Research, Innovation and Social Responsibility, Universidad Privada del Norte (UPN), Trujillo, Peru; ^2^School of Agroindustrial Engineering, Universidad Nacional de Trujillo (UNT), Trujillo, Peru

**Keywords:** red wine, polyphenols, antioxidant, anti-inflammatory, disease prevention, emerging technologies, extraction

## Abstract

There is ample evidence regarding the health benefits of red wine consumption due to its content of phenolic compounds, as an alternative to improve the state of health and prevent various diseases, being the implementation of procedures that allow a greater extraction and stability of phenolic compounds during the elaboration a key aspect. The first part of this review summarizes some studies, mostly at the preclinical level, on the mechanisms by which phenolic compounds act in the human organism, taking advantage of their antioxidant, anti-inflammatory, antitumor, antithrombotic, antiatherogenic, antimicrobial, antiviral, and other activities. Although the migration of grape components into the must/wine occurs during the winemaking process, the application of new technologies may contribute to increasing the content of phenolic compounds in the finished wine. Some of these technologies have been evaluated on an industrial scale, and in some cases, they have been included in the International Code of Oenological Practice by the International Organization of Vine and Wine (OIV). In this sense, the second part of this review deals with the use of these novel technologies that can increase, or at least maintain, the polyphenol content. For example, in the pre-fermentative stage, phenolic extraction can be increased by treating the berries or must with high pressures, pulsed electric fields (PEF), ultrasound (US), e-beam radiation or ozone. At fermentative level, yeasts with high production of pyranoanthocyanins and/or their precursor molecules, low polyphenol absorption, and low anthocyanin-β-glucosidase activity can be used. Whereas, at the post-fermentative level, aging-on-lees (AOL) can contribute to maintaining polyphenol levels, and therefore transmitting health benefits to the consumer.

## Introduction

In recent years, the consumption of foods rich in bioactive compounds, whose intake has been related to protection against certain diseases, has gained relevance. Red wine is one of the foods that has aroused greater interest, and around which several studies have been developed to elucidate the mechanisms of action and its potential benefit to the consumer’s health. Most of the benefits are attributable to phenolic compounds, which are generally classified into flavonoids (anthocyanins, flavanols, flavonols, among others) and non-flavonoids (stilbenes, phenolic acids, among others) (1, 2). These compounds are attributed with antioxidant, anti-inflammatory, antitumor, antithrombotic, antiatherogenic, antimicrobial, and antiviral activity, among other benefits (1–4).

Antioxidant and anti-inflammatory activities are the basis of most of the mechanisms of action involved in the protection of phenolic compounds. Antioxidant activity, observed, for example, in non-alcoholic red wine, by increasing the activity of the catalase, superoxide dismutase (SOD), and glutathione reductase (GR) enzymes (5), or increasing the production of nitric oxide (NO), with a consequent lower cardiovascular risk (6), is particularly interesting.

Quantitatively, the most abundant polyphenols are anthocyanins, which are mainly located in the grape skin and account for 50–60% of the phenolic fraction (7, 8) and flavanols, which are mainly found in the seeds and stalk (8).

Another group of interest, from a qualitative point of view, are the stilbenes, mainly resveratrol (trans-3,4′,5-trihydroxystilbene) and its glycoside, polydatin (9), to which a large part of the protective effects against various diseases are attributed (4, 6, 8, 9).

### Antioxidant and Anti-inflammatory Activities of Phenolic Compounds

Phenolic compounds may prevent oxidative damage caused by free radicals that may cause damage to proteins, carbohydrates, lipids, or nucleic acids (10, 11). Polyphenols can generate less reactive species (12) by capturing unpaired electrons present in free radicals, in addition to chelating Fe and Cu (13), preventing the consequent production of new free radicals.

Other mechanisms of action include the interruption of autooxidation chain reactions, deactivation of singlet oxygen, mitigation of nitrosative stress, activation of antioxidant enzymes, or inhibition of oxidative enzymes (10). The synergy between antioxidant compounds also contributes, e.g., between tannins (14) or between tannins and compounds such as quercetin and resveratrol (15).

The chemical structure has also been shown to influence antioxidant activity. For example, the presence of a greater number of hydroxyl (−OH) groups in epicatechin gallate and epigallocatechin gallate, concerning the non-galloyl tannins (catechin and epicatechin) (14), as well as the presence of −OH and methoxyl (−OCH3) groups in the B-ring of anthocyanins (16).

One of the most studied compounds is resveratrol, which has been shown capacity to inhibit the so-called “oxidative burst” (production of O_2_^–^ and H_2_O_2_), to inhibit the expression of NADPH oxidase, to inhibit the uncoupling of the endothelial nitric oxide synthase (eNOS) (17–19), to regulate catalase, superoxide dismutase (SOD), glutathione peroxidase (GPx), glutathione reductase (GR), glutathione-S-transferase (GST) and NAD (P) H: quinone oxidoreductase 1 (NQO1) activities (20), as well as to induce endogenous antioxidant defenses such as the nuclear factor (erythroid-derived 2)-like 2 (Nrf2) pathway (21). The Nrf2 pathway regulates the expression of inflammatory biomarkers inducible NO synthase, interleukin 6 (IL-6), tumor necrosis factor-alpha (TNF-α) (22), as well as the microglial function and neuroinflammation as in cases of Parkinson’s disease (23).

**FIGURE 1:**
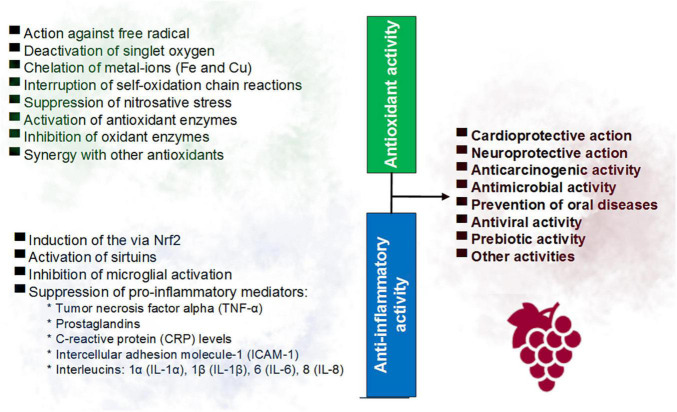
Main involved mechanisms in the antioxidant and anti-inflammatory activities of the phenolic compounds of red wine.

Free radicals such as reactive oxygen species (ROS) can trigger the production of inflammatory mediators such as the cytosine TNF-α, which in turn can lead to increased oxidative stress, in a cycle that contributes to the genesis of pathologies such as cancer, atherosclerosis, neurodegenerative disorders, among others (4, 22, 24). Figure 1 summarizes the main antioxidant and anti-inflammatory activities of red wine polyphenols reported in previous studies.

## Effect of Phenolic Compounds on Specific Pathologies

The following sections summarize the most important mechanisms through which polyphenols exert their protective action against the development of various diseases.

### Cardioprotective Effect

It has been observed that red wine consumption can decrease the level of inflammatory biomarkers related to cardiovascular disease (CVD) such as intercellular adhesion molecule-1 (ICAM-1), IL-6 (25, 26), interleukin 1α (IL-1α) (27) or C-reactive protein (CRP) (26, 28), besides decreasing plasma insulin levels and insulin resistance in patients with high cardiovascular risk and diabetes (29, 30). The latter effects were also observed in non-alcoholic red wine (31). Table 1 summarizes some of the beneficial effects of red wine consumption on biomarkers of cardiovascular risk in various clinical studies.

**TABLE 1:** Some clinical studies on the effect of red wine on biomarkers of cardiovascular risk and related pathologies.

Volunteers (study)	Red wine doses	Main results	References
19 women and 21 men	125 mL/day (women) and 250 mL/day (men)	Higher total antioxidant capacity Higher levels of vitamin E Higher vitamin E/total cholesterol ratio Lower LDL/HDL ratio	(32)
52,367 women and 97,406 men (Paris-Ile-De-France Cohort, France)	Equivalent to 10–30 g/day of ethanol	Higher total antioxidant capacity Higher levels of vitamin E Higher vitamin E/total cholesterol ratio Lower LDL/HDL ratio	(33)
1,896 men (Italian Longitudinal Study on Aging, Italy)	Equivalent to 26.7 g/day of ethanol	Higher HDL levels Higher apolipoprotein Apo A1 levels Lower fibrinogen levels Lower insulin resistance	(34)
10 men (Spain)	Daily doses of 272 mL of red wine, or 272 mL of non-alcoholic red wine, or 100 mL of gin	Lower blood pressure Lower total cholesterol levels Lower triglyceride levels Lower C-reactive protein (CRP) levels Increased probiotic bacteria count	(28)
35 women (Spain)	1 glass of red wine or white wine, equivalent to 20 g/day of ethanol	Higher HDL levels Lower CRP levels Lower levels of intercellular adhesion molecule-1 (ICAM-1) Greater effect with red wine	(26)
23,349 (women and men) (Greek segment of the European Prospective Investigation into Cancer and Nutrition EPIC, Greece)	Equivalent to 10–50 g/day of ethanol in men, and 5–25 g/day in women.	Lower mortality with the Mediterranean diet, with red wine contributing to this effect by 23.5%. Other factors evaluated (low consumption of meat, high consumption of fruits, vegetables, legumes, and monounsaturated lipids), contributed to a lesser degree	(178)

Red wine has also been shown to affect plasma lipoproteins. Proposed mechanisms include decreasing levels of low-density lipoprotein (LDL) or “bad” cholesterol (32), responsible for transporting and depositing cholesterol in tissues (initiation of atherosclerotic plaque formation), and increased levels of high-density lipoprotein (HDL) or “good” cholesterol (33, 34), responsible for removing cholesterol from tissues (35). These benefits have been observed in patients who have previously suffered a myocardial infarction (36) and in patients with carotid atherosclerosis (37).

It is considered that oxidized LDL is responsible for the atherogenic process so that the function of phenolic compounds would be to protect them against oxidation, showing greater activity quercetin and resveratrol, which bind to LDL through glycosidic bonds, protecting them against free radicals and reducing oxidation induced by metal ions (13, 38). Anthocyanins have also been shown to reduce LDL levels, for example, in patients with dyslipidemia (39).

**FIGURE 2:**
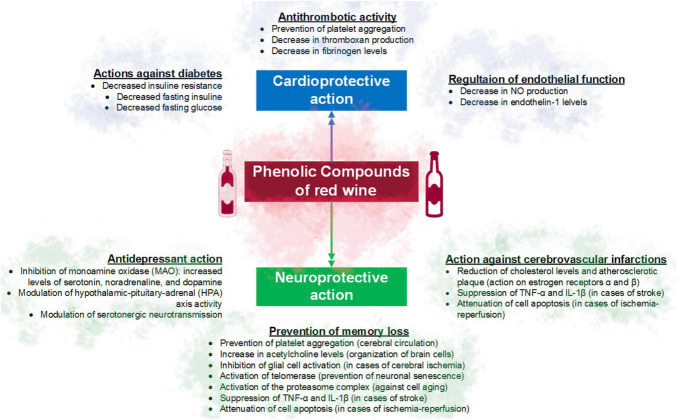
Main cardioprotective and neuroprotective effects of the phenolic compounds of red wine.

Another mechanism includes the effect on nitrous oxide (NO), an important vasodilator, with potential benefits in smokers. Quercetin, tannic acid, malvidin, and resveratrol may contribute to improving endothelial NO production, reducing oxidative stress, vascular inflammation, and platelet aggregation (Figure 2) (40).

### Neuroprotective Effect

Among the main benefits of red wine consumption are the prevention of memory loss, thus attenuating cell death and the development of diseases such as Alzheimer’s disease. The involved mechanisms include the ability of quercetin against cell aging, by activating the proteasome complex (41). Resveratrol has been used to suppress the activation of nuclear transcription factor-kappa B (NF-kB) and the production of prostaglandins (42), reducing the expression of interleukin 1 beta (IL-1β) and TNF-α factor (4, 43), increase the activity of the telomerase enzyme, involved in the prevention of cellular senescence and delay of cognitive decline (44), or promote the activity of sirtuins and peroxisome proliferator-activated receptor-γ co-activator 1-α (PGC-1α) (4, 45). In addition to activating anti-apoptotic Bcl-2 proteins and inactivating pro-apoptotic Bax proteins in the hippocampus (46). Other mechanisms reported in the literature are summarized in Figure 2.

Also, moderate consumption of red wine has been associated with a reduction of up to 50% in the risk of death from stroke (Figure 2) (47) due to the increase in cerebral blood flow, which would be related to the action of resveratrol (48). Resveratrol can also regulate the function of estrogen receptors α and β, reducing cholesterol levels (49) and the formation of atherosclerotic plaque, and thus the risk of ictus due to circulatory failure (50).

Another benefit of red wine consumption is related to its antidepressive effect. Resveratrol, for example, can regulate the monoaminergic system, increasing the levels of serotonin, noradrenaline, and dopamine (51). Likewise, resveratrol, quercetin, ferulic acid, ellagic acid, and proanthocyanidins can modulate the activity of the hypothalamic-pituitary-adrenal axis (HPA axis), as well as serotonergic neurotransmission (52, 53), mechanisms that play an important role against anxiety and depression.

### Anticancer Activity

The main involved mechanisms in the anticancer activity of phenolic compounds are described below.

#### Action Against Oxidative Damage

Anthocyanins and tannins have shown protection against UV-B radiation-induced damage by acting on the free radicals produced (54), besides suppressing the activity of cyclooxygenase-2 (COX-2) induced by this radiation (55). Quercetin has shown activity against the myeloperoxidase (MPO) enzyme induced by UV-B radiation (56), contributing to preventing the development of skin cancer.

On the other hand, during lipid peroxidation, unstable intermediate compounds are generated, which in turn may act as “new toxic messengers” in successive reactions (57). For example, malondialdehyde, that at high plasma concentrations may be considered a prognostic factor in ovarian cancer (58). Red wine consumption may regulate plasma levels of malondialdehyde (30).

#### Interference on Basic Cellular Functions

Resveratrol has shown activity in key stages of carcinogenesis: as an antioxidant and antimutagen in the initiation phase, and as an anti-inflammatory and inhibitor of COX and hydroperoxidase in tumor cells in the promotion phase, besides inducing cell differentiation in the progression phase (59). Resveratrol has also shown activity against the enzyme tank-binding kinase 1 (TBK1), related to carcinogenic inflammatory processes (60), besides reducing the protein methylation related to breast cancer (61) and the ornithine decarboxylase (ODC) activity responsible for the synthesis of polyamines linked to colorectal carcinogenesis (62).

At a clinical level, it has been possible to reduce the proliferation of tumor cells in patients with colon cancer by 5% with doses of 1 g of resveratrol, and it has also been possible to detect resveratrol-3-O-glucuronide, resveratrol-4′-O-glucuronide, resveratrol-3-O-sulfate, resveratrol-4′-O-sulfate, resveratrol sulfate glucuronide, and resveratrol disulfate, which would indicate a joint anticancer action between resveratrol and its derivatives (63).

Proanthocyanidins have been shown to reduce DNA methylation levels and inhibit DNA-methyltransferase and histone deacetylase (HDAC) activities, promoting the re-expression of tumor suppressor genes in skin cancer cells (64). While quercetin has been shown to inhibit the growth of melanoma cells by blocking the transducer protein and inhibiting the activation of signal transducer and activator of transcription 3 (STAT3) proteins (65).

#### Cancer Cell Apoptosis

The induction of cancer cell apoptosis involves mechanisms such as cell cycle stop, blockade of c-Jun N-terminal kinase (JNK), NF-kB factor inhibition, kinase C suppression (66), deregulation of fatty acid synthase (FAS), activation of DAPK2 and BNIP3 genes that encode proapoptotic enzymes (67), among others.

Resveratrol at low doses can be useful for maintaining health, while at high doses it can be useful for inducing cell apoptosis (68), and therefore a therapeutic alternative against cancer.

For its part, the ellagic acid has been shown capacity to stimulate apoptosis in prostate carcinoma (PC3) cells by activating the caspase enzyme, besides a decrease in the levels of antiapoptotic protein Bcl-2 and an increase in the proapoptotic protein Bax (69). Delphinidin has shown apoptotic activity in colon cancer cells (70), in addition to retarding growth of liver cancer cells (71) and PC3 cells (72).

#### Action on Migration, Invasion and Metastasis Processes

Proanthocyanidins can alter migration and invasion processes in pancreatic cancer cells (73) by inhibiting phosphorylation of extracellular signal-regulated kinase 1/2 (ERK1/2) and the inactivation of NF- kB factor. While delphinidin, cyanidin, and resveratrol have shown anti-metastatic activity in human colon cancer cells (74, 75).

The use of resveratrol in synergy with zinc and other compounds for the treatment of prostate cancer has also been suggested (76), which requires further studies to elucidate the action mechanisms, as well as the conditions under which the treatment is clinically effective.

### Prevention of Oral Diseases

Phenolic compounds in red wine have shown effectiveness against microorganisms involved in the development of caries and periodontitis. Proanthocyanidins act on *Streptococcus mutans*, responsible for the formation of dental plaque, by inhibiting the enzyme glucosyltransferase (GTF), which catalyzes the conversion of sucrose into glucans (base of dental plaque) (77), besides inhibiting the enzyme F-ATPase, responsible for protecting *S. mutans* from acidic environmental stress generated in the environment of dental plaque (78). For their part, *Fusobacterium nucleatum*, *Streptococcus oralis*, and *Actinomyces oris* have shown sensitivity to red wine, non-alcoholic red wine, and red wine solutions enriched with grape seed extracts, an effect attributed to catechin and procyanidin B2 from the seeds (79).

Regarding quercetin, it has shown effects against alveolar bone loss in rodents affected by periodontitis, by reducing the production of inflammatory mediators such as IL-1β, IL-17, TNF-α, of the receptor activator of NF-κB ligand (RANKL) and ICAM in gingival tissue (80). Resveratrol has shown a similar effect against oxidative stress and the progression of periodontitis through the activation of sirtuins and the Nrf2 pathway, besides helping to improve alveolar bone resorption (81).

### Prebiotic Effect

An increase in the bacteria *Enterococcus, Bacteroides, Bifidobacterium, Eggerthella tarda, Blautia coccoides*, among others, has been observed in the intestinal microflora after the consumption of red wine and non-alcoholic red wine (28). Similar results were subsequently obtained with *Enterococcus*, *Bifidobacterium*, and *E*. *lenta*, proposing the interaction of anthocyanins with these bacteria, especially with *Bifidobacterium*, whose growth was positively correlated with the presence of syringic and 4-hydroxybenzoic acids, both anthocyanin derivatives (82). Thus, the prebiotic effect of red wine would be related to the catabolism of phenolic compounds.

### Antiviral Activity

The action mechanisms include the antioxidant capacity of phenolic compounds, as well as their capacity to inhibit essential viral enzymes, activate self-defense mechanisms, and prevent the attachment and penetration of the virus into the host cell (83). The latter is of vital importance to inhibiting the synthesis of viral DNA. Quercetin and myricetin have been shown to inhibit DNA and RNA polymerase activity (84), involved in the process of DNA replication and RNA synthesis in viruses (85).

Of special relevance are the recent publications about effect of phenolic compounds against severe acute respiratory syndrome coronavirus 2 (SARS-CoV-2), which causes coronavirus disease 2019 (COVID-19). The tannic acid has shown capacity to inhibit the activity of two proteases that play a key role in cellular entry and replicating of SARS-CoV-2: transmembrane protease serine 2 (TMPRSS2, in the host cell) and 3-chemotrypsin-like protease (M^pro^ or 3CL^pro^, in the virus) (86). At clinical level, quercetin has shown positive effects in the treatment of COVID-19 patients by decreasing the serum levels of alkaline phosphatase (ALP), quantitative C-reactive protein (q-CRP), and lactate dehydrogenase (LDH), as critical markers involved in COVID-19 severity. Besides, earlier discharge time was observed in the patients who were taking quercetin. In general, those patients showed better clinical improvements in terms of the COVID-19 symptoms (87).

The synergistic effect of phenolic compounds with other substances can also be used. Resveratrol can improve the action of decitabine in treatments against human immunodeficiency virus (HIV), by inhibiting the enzyme ribonucleotide reductase, thereby blocking viral DNA synthesis and subsequent virus replication (88). For its part, since cytokine storm and sepsis are major causes of death in severe COVID-19, in patients who had received a combined treatment of resveratrol and copper (preprint: observational study), the number of deaths in resveratrol-Cu group was 1.9-fold lower respect to patients with standard care. Although the mechanism of action is unclear, it could be related to the generation of free radicals, which can inactivate or degrade cell-free chromatin released from dying cells and contributing to the sepsis cascade. However, according to the authors, although these results are promising, these need to be confirmed in a randomized clinical trial (89).

However, the antimicrobial and antiviral activity shown by red wine and/or its constituents cannot be comparable to that shown by antibiotics and antivirals, so it should not be used for this purpose.

## Technological Improvements on the Content of Phenolic Compounds

Various investigations have focused in recent years on increasing the content of phenolic compounds in red wine. Although most of them are aimed at improving the physicochemical stability and the sensory profile, they can also be used to improve the bioactive profile.

It is estimated that around 50% of polyphenols are extracted into the must/wine during vinification (90), that is, almost a half remain in the grape pomace. These polyphenols are not always used, for example, for the development of new foods and other applications (91).

Traditional practices such as the use of pre-fermentative enzymes and cold maceration can help improve the extraction of anthocyanins and tannins (92). On the other hand, the contact time between the skins and the must/wine is an important aspect during the transfer of polyphenols, as in the case of resveratrol, which in some cases reaches its maximum extraction after 10 days (93).

**FIGURE 3:**
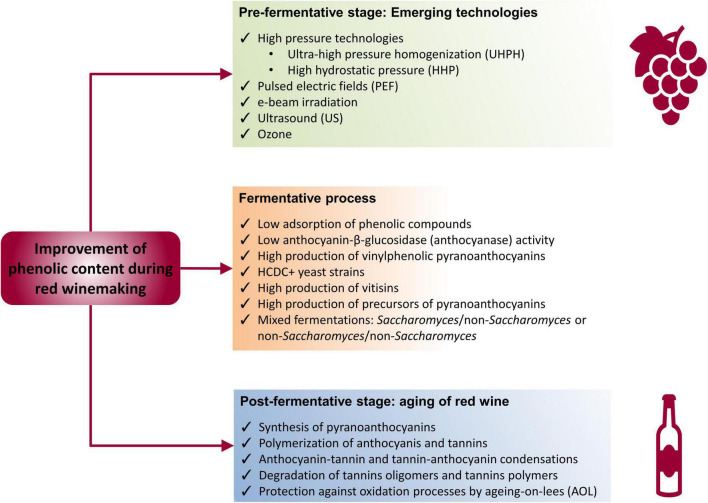
Technological strategies to improve the content of phenolic compounds during red winemaking.

However, the use of non-conventional technologies applicable in the different stages of winemaking process may increase the extraction of polyphenols. These technologies include high pressures, ultrasound (US), pulsed electric fields (PEF), e-beam irradiation, and ozone, all of them applicable in the pre-fermentative stage (in grape berries or grape must), in addition to biotechnological strategies at fermentative level and during the aging of red wine (Figure 3).

### At Pre-fermentative Level

The so-called emerging technologies have been studied mainly for the control of microbial load in food. However, they may also be useful to improve the extraction of phenolic compounds, with the advantage of reducing maceration times, not generating thermal damage to the treated product, inactivating oxidative enzymes, and reducing SO_2_ doses (94). SO_2_ is an additive that has been related to health problems in the consumer (95) and to generate aromatic defects related to its excessive use (96).

Emerging technologies can also be used to improve extraction in grapes with low phenolic content, as a complementary treatment, or as an alternative to the traditional use of pectolytic enzymes or the “blended” with grape varieties with higher phenolic content, allowing, for example, the elaboration of wines with higher levels of resveratrol (up to 16 mg/L in Pinot noir wines) (97) and quercetin (up to 13 mg/L in Shiraz-grape wines) (90).

#### High-Pressure Technologies

High pressures are one of the promising alternatives for the treatment of grapes and/or musts, highlighting high hydrostatic pressure (HHP) and ultra-high pressure homogenization (UHPH), which have been implemented in the development of equipment for industrial applications [more details in Morata et al. (98)].

##### High Hydrostatic Pressure

HHP is a discontinuous flow technique. Besides reducing the microbial load and preserving aroma, HHPs can improve the extraction of phenolic compounds into the must/wine, as well as protect them against oxidation by partially inhibiting the PPO enzyme at pressures greater than 600 MPa (99), which makes it possible to keep the antioxidant properties of wine and reduce the necessary doses of SO_2_.

Another advantage of HHP is that once applied it allows to keep the integrity of the berry (100), thus facilitating the handling of the grape, without loss of raw material or risk of microbial contamination. On the contrary, the diffusion of anthocyanins to the pulp and the external surface of the seeds is facilitated, because of the rupture of the cell walls of the skin (Table 2) (101).

**TABLE 2:** Applications of high-pressure technologies to improve the extraction of phenolic compounds during red winemaking.

High pressure technology	Sample (volume)	Operation parameters	Main results respect to control treatment	Reference
**HHP** Discontinuous process	Red grape berries (Tempranillo) 300 g	200 MPa, 10 min, < 30^°^C	**Treated grape berry:** • Migration of anthocyanins to pulp and seeds • No external modifications of fruit appearance (integrity) **Wine:** •↑ extraction of p-coumarylated anthocyanins at (> 68%) •↑ TPI (> 43%) •↑ CI (> 26%)	(101)
**HHP** Discontinuous process	Red grape by-products	600 MPa, 60 min, 70^°^C	•↑ total phenolic content (> 1.5-fold higher) •↑ extraction of acylated anthocyanins (> 10.7-fold higher) •↑ antioxidant activity (> 2.75-fold higher)	(102)
**HHP** Discontinuous process	Red wine (Agiorgitiko) 1 L	350 MPa, 10 min, 8^°^C The wine was dosed with 30, 60 and 100 mg/L SO_2_	Wines with < 60 mg/L SO_2_ (6 months of storage): •↓ flavanols •↓ monomeric anthocyanins •↓ antioxidant activity	(106)
**UHPH** Continuous process	Red grape must (Cabernet Sauvignon) 100 L at 60 L h^–1^	300 MPa, 77°C, < 0.2 s	**Red grape must:** ↑ protection against oxidation (> 2.5-fold higher) after 6 days of exposure to air (without SO_2_)* **Red wine:** Maintains the total anthocyanins content	(110)

*Oxidative process: monitored by the evolution of the hue (evolution from red-blue to red-brown tonalities).

HHP improves the selective extraction of phenolic compounds, for example, acylated anthocyanins, which would be related to a better solubility of acylated anthocyanins (less polar than the non-acylated ones), due to the effect of HHP on must polarity (decrease in the dielectric constant of water), and the decrease in pH (molecular deprotonation at high pressures), improving the content of *p*-coumaroylated anthocyanins, color intensity (CI), and total polyphenol index (TPI) (101).

A better extraction of polyphenols from red grape residues at 600 MPa has also been observed (Table 2), increasing the extraction of acylated anthocyanins, besides increasing the extraction of phenolic compounds with HHP, US (35 KHz), and PEF (3 kV cm^–1^), and improving the antioxidant capacity with HHP and PEF, regarding the control. In the latter case, it may be related to the inactivation of oxidative enzymes (102).

On the other hand, HHPs can favor the formation of pyranoanthocyanins, mainly derivatives of vitisin A at 600 MPa and 70^°^C (103), while at 500 MPa (5 min at 20^°^C) levels of pyranoanthocyanins, prodelphinidins, and polymerized tannins similar to those found in samples obtained with micro-oxygenation and in contact with wood, have been observed (104). However, the content of anthocyanins such as cyanidin may decrease, as occurs with other techniques such as e-beam irradiation (101, 105), thus being able to affect the antioxidant activity.

HHP has also been applied on wines to maintain stability and reduce the necessary doses of SO_2_. However, a decrease in the content of aromatic compounds, phenolic compounds, and antioxidant capacity has been reported in red wines stored for 12 months and treated with HHP at 350 MPa, in which it is necessary to keep a minimum SO_2_ concentration of 60 mg/L (Table 2) (106). So that, HHP can be applied as a complementary treatment to reduce the necessary doses of SO_2_.

##### Ultra-High Pressure Homogenization

UHPH is a technology that is arousing interest in winemaking, mainly for microbial inactivation and inactivation of oxidative enzymes in white musts (107–109), acceleration of yeast autolysis, and production of yeast derivatives for oenological use (108). However, few publications have addressed their applicability in red winemaking processes.

UHPH is a continuous flow technique applicable to liquid samples, for example, musts, at pressures between 200 and 600 MPa, followed by instantaneous depressurization to atmospheric pressure with the consequent generation of 100–300 nm nanofragmentations in the treated sample (94). It is characterized by having a total process time of 0.2 s, with the advantage of not generating damage at the level of vitamins, aromatic compounds, and pigments, even at temperatures close to 80^°^C (94, 107, 110). Another advantage of UHPH is a greater release of nitrogenous compounds for the nutrition of the yeast in the treated musts, besides allowing the synthesis of fermentative esters with a positive impact on the aroma of the wine compared to the conventional treatment with SO_2_ (109).

The application of UHPH allows an almost complete inactivation of the enzyme polyphenoloxidase (PPO) (> 90%) (107, 109), with the consequent protection of phenolic compounds, besides maintaining the antioxidant capacity of the must, and color stability, even when exposed to air (Table 2) (110), being greater its capacity to inactivate oxidative enzymes concerning high hydrostatic pressure (HHP).

Although it has not been reported in the literature the effect of UHPH on other grape enzymes with a direct impact on wine quality, such as β-glucosidase, β-lyase, protease, pectinase, hydroxycinnamate decarboxylase (HCDC), cellulase, among others (111, 112), the effect of UHPH at the level of quaternary structure denaturation in proteins should be considered (113). However, the use of exogenous enzymes can help counteract the effect of UHPH on these grape enzymes.

Unlike HHP, which works under static conditions, high-pressure homogenization is a dynamic technology, and its effect, besides high pressure, depends on phenomena such as cavitation, turbulence, and shearing (108). Initially, UHPH would not be applicable for the processing of red grape mash due to the presence of peels and seeds, which can clog the homogenizer valves, considering that particle sizes smaller than 500 μm have been suggested (94). Thus, the application of UHPH would be limited only to musts free of peels, seeds, and other solids. Previously, the application of high-pressure homogenization, at 200 MPa at a flow rate of 120 L h^–1^, to Trepat red grape must has been reported, in order to reduce the microbial load and evaluate the impact on the sensory profile. However, the article does not mention whether the red must was treated together with seeds and peels (grape mash), or whether these solids were previously separated (114).

Considering that one of the main advantages of UHPH is its inhibitory effect on PPO (107, 109, 110), an interesting alternative has recently been reported in Cabernet Sauvignon red must (110), which, after a maceration process at 0^°^C for 15 days, was separated from peels and seeds, and subsequently treated with UHPH at 300 MPa (Table 2). Among the most important results, the maintenance of anthocyanin levels in the wine produced stands out.

Another alternative could include the application of technologies that allow greater extraction of phenolic compounds during maceration from the grape mash, such as ultrasound, pulsed electric fields, irradiation, or ozone, following the methodology developed by Vaquero et al. (110), with the subsequent draining of the must to separate seeds and peels, followed by the application of UHPH to reduce the microbial load and inactivate the PPO, protecting the polyphenols against oxidation.

#### Pulsed Electric Fields

Through Resolution OIV-OENO 634-2020, the International Organization of Vine and Wine (OIV) included the use of pulsed electric fields (PEF) for treatment of red grapes destemmed and crushed in order to facilitate and increase the extraction of polyphenols and other valuable substances located inside the grape cells and reduce maceration time. The text has been included in the International Code of Oenological Practices.

Several works have reported the application of pulsed electric fields (PEF) to reduce the microbial load in winemaking processes, in which their efficacy for the extraction of polyphenols is also highlighted (Table 3). Among the advantages of PEFs, the reduction of SO_2_ doses during must sterilization stands out (115), besides improving the extraction of phenolic compounds and preserving color at doses of up to 41 kV cm^–1^ (116).

**TABLE 3:** Applications of pulsed electric fields to improve the extraction of phenolic compounds during red winemaking.

Technology	Sample (volume)	Operation parameters	Main results respect to control treatment	References
**PEF** Discontinuous process	Red grape must (Garnacha, Mazuelo, and Graciano) 25 g	2–10 kV cm^–1^, 0.4–6.7 kJ kg^–1^, 50 pulses, 1 Hz, < 30^°^C	**Must from three varieties:** Extraction of anthocyanins and total phenols increase as PEF intensity increases **Mazuelo grape must:** Maximum extraction at 120 h (10 kV cm^–1^): • Anthocyanins: 41.8% higher than control • Total phenols: 31% higher than control **Red wines** (Garnacha, **Mazuelo,** Graciano): • Anthocyanins: 11.3, **16.3**, 7.6% higher control, respectively • Total phenols: 14.2, **14.2**, 12.1% higher control, respectively	(117)
**PEF** Continuous process	Red grape must (Cabernet Sauvignon) 118 kg h^–1^	2–7 kV cm^–1^, 0.56–6.76 kJ kg^–1^, 50 pulses, pulse width 3 μs, 0.41 s, 122 Hz, < 23°C	**Must/wine:** Maximum extraction at 96 h (5 kV cm^–1^): • Anthocyanins: 40% higher than control • Total phenols: 30% higher than control	(179)
**PEF** Continuous process	Red grape must (Garnacha) 4,500 kg at 1,900 kg h^–1^	4 kV cm^–1^, 1.5 kJ kg^–1^, 20 pulses, pulse width 3 μs, 60 μs, 0.41 s, 250 Hz	**Must/wine:** After 7 days of maceration: • Color intensity: 12.5% higher • Anthocyanin content: 25% higher • Polyphenol index: 23.5% higher	(174)
**PEF** Discontinuous process	Red grape must (Pinot Noir) 200 g	1.5 kV cm^–1^, 14.48 and 69.99 kJ kg^–1^, 243 and 1,033 pulses, pulse width 20 μs, 50 Hz, < 25^°^C	• Maximum extraction of malvidin-3-O-glucoside (224% higher) after 8 days of maceration • Maximum extraction of total phenols (61% higher) after 4 days of maceration • Bioprotective capacity of Caco-2 cells against H_2_O_2_ exposure (> 25% for cell viability) after 8 days of maceration	(124)
**PEF** Discontinuous process	Red grape must (Pinot Noir and Merlot) 5 kg	7 kV cm^–1^, 178 Hz	**Grape must (Merlot/Pinot Noir):** • Total phenols: 1.54- and 3.16-fold higher, respectively • Total flavonoids: 1.42- and 4.96-fold higher, respectively • Monomeric anthocyanins: 1.14- and 2.35-fold higher, respectively • Antioxidant capacity: up to 5.93- and 3.90-fold higher, respectively **Red wine (Merlot/Pinot Noir):** • Total phenols: 1.72- and 2.98-fold higher, respectively • Total flavonoids: 2.66- and 6.21-fold higher, respectively • Monomeric anthocyanins: 1.05- and 1.11-fold higher, respectively • Antioxidant capacity: up to 2.18- and 5.77-fold higher, respectively	(180)
**PEF** Continuous process	Red grape must (Grenache) 600 kg h^–1^	0.7–7.8 kV cm^–1^, 78–5,000 μs, 12–290 Hz	**Red wines from PEF treated musts** **Fresh fermented wine/after 12 months of bottle storage** • Anthocyanins: up to 61%/44% higher, respectively • Condensed tannins: up to 30%/10% higher, respectively Reduction of maceration time up to 37%	(118)
**PEF** Continuous process	Red grape must (Merlot) 500 kg h^–1^	33.1–41.5 kV cm^–1^, 4.7–49.4 kJ L^–1^, 2–25 Hz	**Grape must:** •↑ total phenolic content (23–162% higher) •↑ malvidin-3-O-glucoside (17–636% higher) **Must/wine:** Equivalent contents of malvidin-3-O-glucoside and phenolic content at: • PEF-treated: after 1 day of cold maceration • PEF-untreated: after 4 days of cold maceration	(116)
**PEF** Continuous process	Red grape must (Sangiovese) 50 kg at 200–300 L h^–1^	0.9–3.0 kV cm^–1^, 10.4–32.5 kJ kg^–1^, 712–1,069 pulses, 0.48–0.71 s, 1,500 Hz	Must*: • PEF: ↑ total phenolic content (25%) • PEF + dm: ↑ total phenolic content (27%) • PEF + dm + sm: ↑ total phenolic content (42%) **Wine from PEF + dm + sm musts** (3 months of storage)**:** •↑ total phenolic content (40%) •↑ Fe-reactive polyphenols (29%) •↑ tannins (61%) •↑ protection against oxidation (> 1.4-fold higher) **	(130)
**PEF** Continuous process	Red grape must (Rondinella) 200 kg at 250 L h^–1^ Rondinella: low-color red grape	1.5 kV cm^–1^, 2–20 kJ L^–1^, pulse width 3 μs, 400 Hz	**Red wine from PEF treated must (10 and 20 kJ kg**^–^**^1^)** **2 months of bottle storage** •↑ content of anthocyanins (32 and 36% higher, respectively) •↑ content of tannins (64 and 64% higher, respectively) **12 months of bottle storage** •↑ content of anthocyanins (3.8 and 50% higher, respectively) •↑ content of tannins (38 and 50% higher, respectively)	(129)
**PEF** Continuous process	Red grape must (Grenache) 12,000 kg at 2,500 kg h^–1^	4.3 kV cm^–1^, 6.2 kJ kg^–1^, 3.7 square pulses of 100 μs	**Fresh fermented wine** • Anthocyanins: up to 37% higher • Tannins content: up to 40% higher **24 months of bottle aging** • Total anthocyanins: up to 110% higher • Total hydrocinnamic acids: up to 167% higher • Total flavonols: up to 200% higher • Total flavanols: up to 28% higher **6 months of oak barrel aging + 18 months of bottle aging** • Total anthocyanins: up to 81% higher • Total hydrocinnamic acids: up to 152% higher • Total flavonols: up to 189% higher • Total flavanols: up to 96% higher	(128)
**PEF** Continuous process	Red grape must (Grenache) 100 kg at 120 kg h^–1^	5–17.5 kV cm^–1^, 63.4–115 kJ kg^–1^, 45 pulses, 0.38–0.24 s, 120 kg/h	**Red wine fermented by non-*Saccharomyces* yeasts** Depending on the used yeast-strain: •↑ content of pyranoanthocyanins (up to 30% higher) •↑ content of vitisin A (up to 89% higher)	(115)
**PEF** Discontinuous process	Red grape pomace	3 kV cm^–1^, 10 kJ kg^–1^, 30 pulses, 15 s, 2 Hz, 70^°^C	•↑ total phenolic content (> 1.5-fold higher) •↑ extraction of anthocyanin monoglucosides (> 1.4-fold higher) •↑ extraction of acylated anthocyanins (> 7.9-fold higher) •↑ antioxidant activity (> 4-fold higher)	(102)

*Results respect to control for each treatment (without PEF application). dm, dynamic maceration 2 h; sm, static maceration 12 h.

** Oxidative process: monitored by the evolution of the hue (evolution from red-blue to red-brown tonalities).

This technology has shown high efficiency in the extraction of phenolic compounds, at rates higher than 50% (102), due to its action on the cell walls of the skin, producing fragmentation at the nanometric scale, besides reducing maceration time up to 50% at doses of 5–10 kV cm^–1^ (117) and 75% at doses of 33–41 kV cm^–1^ (116), being the efficacy variable depending on the intensity of the PEF, duration of the pulse or number of pulses (118), as well as the variety of treated grape (117).

The application of PEF (0.8 kV cm^–1^, 100 ms, 42 kJ kg^–1^ and 5 kV cm^–1^, 1 ms, 53 kJ kg^–1^, respectively) in Cabernet Franc musts (119) has allowed obtain higher yields of: anthocyanins (46 and 62%, respectively, concerning the control) and tannins (50 and 59%, respectively), besides increasing the antioxidant capacity (51 and 52%, respectively) at the end of fermentation. In a similar study with Cabernet Franc and Cabernet Sauvignon musts (120), the application of PEF (5 kV cm^–1^, 1 ms) during cold maceration improved the extraction of quercetin 3-β-D-glucoside (100 and 74%, respectively, concerning the control), anthocyanins (98 and 60%, respectively) and proanthocyanidins by more than 35% in both varieties, in addition to increasing antioxidant activity by more than 100%.

Subsequently, the application of PEF (0.8 kV cm^–1^, 100 ms and 5 kV cm^–1^, 1 ms) during cold maceration and alcoholic fermentation in Cabernet Sauvignon musts was studied, obtaining a better extraction of anthocyanins and proanthocyanidins in the maceration stage with 0.8 kV cm^–1^ (121).

Furthermore, the application of PEF (7.4 kV cm^–1^) to Tempranillo, Graciano, and Grenache musts (122) improved anthocyanin extraction by up to 49, 87, and 163%, respectively, concerning the control. However, the most remarkable feature of this study was the improvement of total stilbene content, up to 60, 200, and 50%, respectively, mainly the *trans*- and *cis*-piceido fractions (glycosidic forms of resveratrol). No significant effect was observed in the extraction of *trans*-resveratrol, which would be related to the absence of ethanol in the maceration stage (123).

Regarding the antioxidant capacity, improvements of more than 25% were obtained in the viability of Caco-2 cells incubated in digested grape must, against H_2_O_2_-induced stress. The digested grape must was obtained by digestion of Pinot Noir must treated with PEF (1.5 kV cm^–1^) and macerated for 8 days (Table 3) (124).

PEFs can also increase the selective extraction of acylated anthocyanins by more than 7 times at a dose of 3 kV cm^–1^ in comparison to the control (Table 3) (102), besides contributing to the depolymerization of the skin tannins, improving the permeability and diffusion through the cell walls fragmented by the PEF (4 kV cm^–1^, 1 ms) (125). Although from a sensory point of view smaller tannins can increase the sensation of astringency and bitterness in the wine (125, 126), the application of processes such as aging-on-lees may help reduce their impact.

Respect to aging of wine, a higher content of flavanols, flavonols, anthocyanins, and hydroxycinnamic acids has also been reported after 12 and 24 months of aging in Cabernet Sauvignon and Grenache wines (respectively) made from grapes treated with PEF at doses of 5 and 4.3 kV cm^–1^, respectively (Table 3) (127, 128). Intensities lower than 4 kV cm^–1^ followed by macerations for 6 days allowed higher anthocyanin and tannin extractions than treatments at intensities > 5.5 kV cm^–1^ with 4-day macerations (Table 3) (118), conditions that could be applied to reduce maceration time and improve phenolic content and other parameters of red wine at the end of the fermentation and aging process.

PEFs also improve the extraction of anthocyanins and total phenols in grape varieties considered to have a low red color, such as Rondinella, being possible to increase anthocyanin contents by more than 50% in comparison to wines made with untreated must, and by more than 30% in comparison to wines made with musts treated with pectolytic enzymes (129). An alternative to improve the extraction of polyphenols is the combined application of PEF and pre-fermentative maceration (immediately after destemming and crushing the grapes), in order to obtain a higher content of polyphenols and a greater antioxidant capacity (130).

#### Ultrasound

Ultrasound (US) is a technology that can be used for the extraction of phenolic compounds thanks to the mechanical action that produces the successive compression and expansion of the bubbles formed by the ultrasonic waves, which, when collapsing, release energy, reaching localized temperatures of up to 5,000 K and pressures up to 200 MPa (131).

Through Resolution OIV-OENO 616-2019, the International Organization of Vine and Wine (OIV) included the use of US during pre-fermentative maceration, after destemming and crushing, to improve the extraction of phenolic compounds, besides shortening the maceration time and limiting the excessive extraction of tannins in grapes with deficient phenolic maturation. The text has been included in the International Code of Oenological Practices.

The treatment of red musts with US is an effective alternative to increase the extraction of tannins and total phenols by up to 58 and 27%, respectively (132), and to maintain anthocyanin levels by up to 97% (133), without affecting its chemical stability. The treatment with US may also generate a greater increase in the content of polymeric anthocyanins in wines stored for 12 months (132) compared to the increase observed in the same fresh fermented wines (at the end of alcoholic fermentation) (Table 4).

**TABLE 4:** Applications of ultrasound and irradiation to improve the extraction of phenolic compounds during red winemaking.

Technology	Sample (volume)	Operation parameters	Main results respect to control treatment	Reference
**US**	Red grape berries (Monastrel) 800 g	40 Hz, 280 W, 90 min, 18°C	**Fresh fermented red wine: US alone and sequential enzyme + US** • Total phenols: 9 and 28% higher, respectively • Total tannins: 19 and 42% higher, respectively • Anthocyanins: 7.5 and 13% higher, respectively • Polymeric anthocyanins: 9 and 31% higher, respectively **Red wine 3 months of bottle storage: US alone and sequential enzyme + US** • Total phenols: 12.5 and 27% higher, respectively • Total tannins: 14 and 59% higher, respectively • Anthocyanins: 9.5 and 9.5% higher, respectively • Polymeric anthocyanins: 13 and 50% higher, respectively	(134)
**US**	Red grape berries (Tempranillo and Monastrell) 400 kg at 400 kg h^–1^	28 kHz, 2,500 W, 8 W cm^–2^	**Tempranillo red wine (fresh fermented and 5 months of bottle storage):** • Total phenols: 12 and 9% higher, respectively • Anthocyanins: 18 and 48% higher, respectively **Monastrell red wine (fresh fermented and 12 months of bottle storage):** • Total phenols: 27 and 34% higher, respectively • Total tannins: 58 and 46% higher, respectively • Polymeric anthocyanins: 12 and 21% higher, respectively	(132)
**US**	Red grape pomace	35 KHz, 60 min, 70^°^C	•↑ total phenolic content (> 1.5-fold higher) •↑ extraction of acylated anthocyanins (> 6-fold higher) •↑ antioxidant activity (> 1.5-fold higher)	(102)
**US**	Red grape pomace (Tannat)	Bath mode: 50–100 W, 50 min, 30^°^C	**Extraction in fresh/freeze-dried pomace** • Total phenols (100%/180% higher, respectively) • Total monomeric anthocyanins (100%/180% higher, respectively) • Antioxidant capacity (180% higher in freeze-dried)	(181)
**γ -irradiation**	Red grape berries (Cabernet sauvignon and Shiraz) 500 g	0.5–2 kGy	**Better results: wines from irradiated grapes at 1.5 kGy:** Cabernet Sauvignon wines (fresh and 4 months of bottle storage) • Total anthocyanin content: 97 and 77% higher, respectively • Total phenolic content: 23 and 31% higher, respectively • Total antioxidant capacity: 42 and 37% higher, respectively Shiraz wines (fresh and 4 months of bottle storage) • Total anthocyanin content: 29 and 45% higher, respectively • Total phenolic content: 16 and 18% higher, respectively • Total antioxidant capacity: 21 and 19% higher, respectively	(137)
**e-beam irradiation** Continuous process	Red grape berries (Tempranillo) 700 g	0.5–10 kGy, 10 MeV, 50 kW, 100 Hz	• **Grape berry:** No external modifications of appearance (integrity) • **Grape must:** Total anthocyanins 70% higher at 10 kGy • **Wine:** Vinylphenolic pyranoanthocyanins and vitisins were more stable	(136)
**Ozone**	Red grape berries (Pignola) 6 kg Red grape subject to dehydration for wine production	**Shock treatment:** 1.5 g/h of O_3_ for 18 h followed by dehydration in normal atmosphere by 24 days	**Grape must after 24 days of dehydration:** • Total phenolic content: 16% higher • Anthocyanins: 14% lower	(142)
		**Long exposure:** 1.5 g/h of O_3_ for 18 h followed by dehydration in normal atmosphere with 0.5 g/h of O_3_ for 4 h each day by 22 days	**Grape must after 22 days of dehydration:** • Total phenolic content: 67% lower • Anthocyanins: 14% lower	
**Ozone**	Red grape berries (Petit Verdot) 500 kg	1.2 g/h of O_3_ for 12 h at 4^°^C	**Grape must:** • Anthocyanins: 19% higher • Tannins: 16% higher **During fermentative process:** Maximum extraction of grapes O_3_-treated and untreated: • Anthocyanins: 5 and 11 days, respectively • Phenolics: 7 and 16 days, respectively **Wine after malolactic fermentation and stabilization** Anthocyanins: 14% higher	(140)
**Ozone**	Red grape berries (Nebbiolo) 500 kg	30 μL/L at 20^°^C	**Extraction yields in must of Nebbiolo at the end of maceration (240 h)** Anthocyanins: • 24 h O_3_-treated grapes: 87% • 72 h O_3_-treated grapes: 64% • Control (no-treated grapes): 60% Oligomeric flavanols: • 24 h O_3_-treated grapes: 86% • 72 h O_3_-treated grapes: 91% • Control (no-treated grapes): 79% Polymeric flavanols: • 24 h O_3_-treated grapes: 89% • 72 h O_3_-treated grapes: 90% Control (no-treated grapes): 81%	(141)

It is also possible to take advantage of the application of US together with other treatments (Table 4), for example, in sequential applications with pectolytic enzymes (134). Although the decrease in monomeric anthocyanins and the increase in polymeric forms is a normal phenomenon during red wine aging, the treatment of musts with US has made it possible to obtain red wines (after 3 months of bottle storage) with a higher content of polymeric anthocyanins and tannins compared to the control wine produced through the traditional process (134). The increase in these phenolic compounds was even greater when the musts were treated sequentially with pectolytic enzymes and US. Although in both cases a decrease in the monomeric anthocyanin content was reported, the application of US to the must reduced the loss of anthocyanins in the elaborated wine.

Like other technologies, the efficiency in the extraction of phenolic compounds depends on the intensity of US. For example, an optimal range between 12 and 37.5 kHz has been reported to increase anthocyanin extraction by up to 18%. Higher doses can alter the content of anthocyanins (135).

#### Electron Beam Irradiation

Electron beam irradiation (e-beam) is characterized by the use of high-energy accelerated electrons and can be applied continuously on an industrial scale without increasing the temperature of the treated product, even at doses of 10 kGy (100), with the advantage of reducing the necessary doses of SO_2_ as a preservative and antioxidant.

Doses of 10 kGy may improve the extraction of anthocyanins by more than 70% (Table 4) (136), also improving the fruity smell of the produced wine. Doses of less than 10 kGy affect the pectin structure, allowing a greater diffusion of phenolic compounds into the must/wine, besides reducing the necessary doses of exogenous pectins (98). Lower doses (0.5–3 kGy) have also shown improvements in the extraction of anthocyanins and phenolic compounds in general (137, 138), and doses of 2 kGy have allowed obtaining better antioxidant capacity in wines made from irradiated grapes (137).

A recent study has reported the decrease in the content of flavonols, flavanols, anthocyanins, and phenolic acids in red wines treated directly with e-beam radiation, at doses between 1 and 10 kGy, as an alternative to the use of SO_2_ (139). However, another study reported a higher content of flavonols, flavanols, anthocyanins, total phenols, and higher antioxidant capacity in wines made from grapes irradiated at a dose of 1.5 kGy, compared to wines made from non-irradiated grapes (137).

Furthermore, although the content of phenolic compounds, mainly anthocyanins, decreases during aging, it has been reported that after 4 months in bottle, red wines made from irradiated grapes maintain anthocyanins, total phenols, and antioxidant capacity at higher levels than the respective wines made from non-irradiated grapes (Table 4) (137). Likewise, greater stability of vinylphenolic pyranoanthocyanins and vitisins has been reported in wines made from irradiated grapes at doses of 10 kGy (136). Which indicates that the advantage of this technology would be focused on its applicability on the raw material (grape berry).

#### Ozono

It has been possible to increase the extraction of anthocyanins and tannins by up to 19 and 16%, respectively, in Petit Verdot grapes treated with ozone, besides shortening the fermentation time in large-scale vinifications (Table 4) (140). On the other hand, has been showed higher extractability of flavanols in musts from Nebbiolo grapes treated with ozone (short exposures: < 72 h, 30 μL L^–1^) during maceration (141).

One aspect to optimize is the treatment time, considering that prolonged exposure, even at low concentrations, can induce a decrease in the total phenolic content due to oxidation caused by ozone activity (141, 142). However, short treatments may increase the levels of anthocyanins and tannins in the berry, as a response to mild to moderate ozone-induced stress, activating the protective antioxidant response through the synthesis of phenolic substances (140, 141).

Moreover, the prolonged exposure can harden the skin of the berries (143), which would be related to a reduction of the disassembly phenomena of the pectic structure of the cell wall (less solubilization of pectins, less depolymerization of polyuronides, and less pectin methylesterase activity) (144), generating a slow extraction of polyphenols, for example, of high-molecular-weight flavanols, whose affinity with cell walls increases when the berry is treated with ozone (141). On the other hand, in shorter ozone treatments, higher polygalacturonase and pectin methylesterase activity has been observed (142), whose action contributes to degrading the pectins of cell walls, facilitating the diffusion of polyphenols into the must/wine. The effect of ozone on the hardening of the cell wall of treated berries, and, therefore, on the extractability of polyphenols, also depends on the stage of grape ripeness, ozone concentration (140), and grape variety (Table 4) (141).

During maceration, di-substituted anthocyanins (cyanidin-3-glucoside and peonidin-3-glucoside) are the first ones to migrate into the must, followed by tri-substituted anthocyanins (malvidin-3-glucoside, delphinidin-3-glucoside, petunidin-3-glucoside) (145). It has been proposed that the hardening of ozone-treated berries may result in slower extraction of di-substituted anthocyanins, which are also more easily lost due to oxidation during the early stages of winemaking when oxidative enzymes are more active and dissolved oxygen levels are higher. That is, the slow extraction would help to maintain the content of these anthocyanins at the end of maceration, especially peonidin-3-glucoside (141). In this regard, higher contents of peonidin-3-glucoside (di-substituted) and malvidin-3-glucoside (tri-substituted) have been reported at the end of maceration of Barbera and Nebbiolo grape musts treated with ozone (141). In other words, the application of ozone would make it possible to maintain the final levels of anthocyanins (143).

Finally, the application of ozone would be recommended only on the raw material, that is, in the grape before crushing and destemming, considering that the direct application to red wine at a dose of 3.5 g/h, as an alternative to the use of SO_2_, may lead to the loss of flavonols, flavanols, anthocyanins and phenolic acids (139), thus affecting the bioactive profile of the wine.

### Strategies at Fermentative Level

During alcoholic fermentation, one aspect to consider is the use of yeasts with low expression of anthocyanin-β-glucosidase (anthocyanase) activity, which causes anthocyanin hydrolysis (146), although this enzyme can contribute to improving the varietal aroma of the wine through the release of aromatic compounds from non-aromatic precursors of the grape (112). The use of yeasts with low adsorption capacity for phenolic compounds, such as anthocyanins (126) and resveratrol (147), should also be taken into account, due to their adsorption by the cell walls of yeasts, which due to their non-polar character, have shown greater adsorption capacity for acylated anthocyanins (from higher to lower adsorption: coumarilated > caffeinated > acetylated) compared to the non-acylated ones (148, 149), indicating an inverse relationship between the polarity of anthocyanins and their adsorption. This would also explain the adsorption of resveratrol (with low hydrophilic character) (123, 150), by the cell walls of yeasts.

The adsorption capacity has also been related to the degree of methoxylation and hydroxylation of the B-ring of anthocyanins. Malvidin and peonidin, with a higher degree of methoxylation (more non-polar), are adsorbed to a greater extent than delphinidin and petunidin, with a higher degree of hydroxylation (more polar) (148). On the contrary, a lower adsorption capacity has been observed with vitisins A and B (149).

It is estimated that some *Saccharomyces* strains can adsorb up to 5.8% of anthocyanins (149), while the use of certain non-*Saccharomyces* yeast strains, such as *Metschnikowia pulcherrima* and *Lachancea thermotolerans* can contribute to retaining a higher amount of anthocyanins in wine (151). Therefore, the selection of yeast strains with low anthocyanin adsorption and low anthocyanin-β-glucosidase activity is of utmost importance, in addition to other characteristics such as high production of pyranoanthocyanins.

#### Increased Content of Pyranoanthocyanins

Although many of the technologies applicable to the grapes, must, or even wine, may lead to losses in the content of anthocyanins, at the fermentation level the content of more stable polyphenols may be increased (Table 5). Vinylphenolic pyranoanthocyanins are formed by the condensation of vinylphenols and anthocyanins. The use of yeasts with hydroxycinnamate decarboxylase (HCDC +) activity is an interesting strategy to increase vinylphenolic pyranoanthocyanins in wine since they can release precursor vinylphenols from grape hydroxynamic acids (152). For their part, vitisins A and B come from the condensation of malvidin-3-O-glucoside (M3G) with pyruvic acid and acetaldehyde, respectively, during the fermentation process or aging (153). These pyranoanthocyanins have shown high chemical stability due to the presence of the fourth heteroaromatic ring in their structure, formed from the integration of a non-anthocyanin molecule (vinylphenols, pyruvate, or acetaldehyde) in the structure of the precursor anthocyanin (152), resulting in greater resistance to oxidation.

**TABLE 5:** Strategies applied at the fermentative level and during the aging of red wines to improve and/or maintain the content of phenolic compounds.

Winemaking stage	Technological strategy	Wine	Main results respect to control treatment	References
**Fermentative stage**	**Mixed fermentation** *S. cerevisiae* + *Schizosaccharomyces pombe* and *S. cerevisiae* + *Torulaspora delbruecki*	Syrah (70 mL)	*S. cerevisiae* + *S. pombe* ↑ Total anthocyanins (up to 1.2-fold higher) ↑ Vitisins (up to 1.75-fold higher) *S. cerevisiae* + *Torulaspora delbruecki* ↑ Total anthocyanins (up to 1.4-fold higher) ↑ Vitisins (up to 1.2-fold higher)	(161)
	**Sequential fermentation** *Lachancea thermotolerans* + *Schizosaccharomyces pombe* and *Lachancea thermotolerans* + *S. cerevisiae*	Tempranillo (5 L)	↑ Total anthocyanins (> 1.6 respect to AF + MLF) ↑ Vitisins A and B (> 1.5 and > 2.6, respectively, respect to AF + MLF)	(182)
	Addition of mannoprotein before fermentation	Cabernet Sauvignon (100 L)	•↑ Total polyphenols content (up to 1.2-fold higher) •↑ Total tannin content (up to 3.4-fold higher) •↑ Proanthocyanidin B1 and B2 (up to 1.13- and 1.17-fold higher, respectively) • Maintains petunidin-3-5-O-diglucoside and malvidin-3-5-O-diglucoside levels. •↑ Antioxidant capacity (up to 1.18-fold higher)	(171)
**Wine aging stage**	Application of AOL	Tempranillo (2 L)	Maintains the total anthocyanins content	(183)
	Addition of mannoprotein during storage	Cabernet Sauvignon (100 L)	↑ Cyanidin-3,5-O-diglucoside (up to 10.8-fold higher) ↑ Petunidin-3,5-O-diglucoside (up to 5.4-fold higher)	(171)

*AF + MLF: traditional winemaking process involving alcoholic fermentation (AF) + malolactic fermentation (MLF).*

The synthesis of vinylphenolic pyranoanthocyanidins also contributes to improving anthocyanin protection against degradation by microorganisms such as *Dekkera/Brettanomyces* (154, 155), since this yeast cannot degrade these pyranoanthocyanins (156), allowing their content to be maintained in the wine.

Pyranoanthocyanins have shown antioxidant and anti-inflammatory activity against prooxidant (H_2_O_2_) and pro-inflammatory (TNF-α) molecules, besides neutralizing the secretion of inflammatory biomarkers such as interleukin 8 (IL-8) in human colon adenocarcinoma cell cultures (24). Vitisin A has shown a protective effect against the secretion of the biomarker monocytic chemoattractant protein-1 (MCP-1) induced by the TNF-α factor in human endothelial cell cultures (157). Furthermore, vitisin A has shown good stability under simulated gastrointestinal conditions (*in vitro*) (158).

An increase in the content of vinylphenolic pyranoanthocyanins has been reported in mixed fermentation of *S. cerevisiae* with *Pichia guillermondii* (159), and the synthesis of vitisins in mixed fermentations of *S. cerevisiae* with *Schizosaccharomyces pombe* and with *Torulaspora delbruecki* (Table 5) (160, 161). Species with high acetaldehyde production such as *Saccharomycodes ludwigii* (162, 163) can also be used to increase the synthesis of vitisin B.

Another alternative could be the use of metabolic inhibitors during alcoholic fermentation, to increase the production of acetaldehyde by S. *cerevisiae* (164, 165), because of the inhibitory effect on the enzyme alcohol dehydrogenase (ADH), responsible for converting the aldehyde into ethanol (166), which could increase the substrate available for the synthesis of vitisin B.

### Strategies at Post-fermentative Level

#### Traditional Aging Wines

During aging, a series of physicochemical processes occur that, individually or together, may modify the content of bioactive compounds, because of isomerization, hydrolysis, polymerization, condensation, or even degradation reactions that may have effects on the bioavailability and bioactivity of polyphenols. In general, the content of anthocyanins, resveratrol, and flavonols tends to decrease (7, 126, 167), as a consequence of their transformation into other molecules, so that greater health benefits from consuming young red wines would be expected.

On the contrary, the content of monomeric flavanols may increase due to the hydrolysis of tannins (7). These monomers have shown high antioxidant capacity (13, 14), as well as antimicrobial activity (79). Although from a sensory point of view monomeric tannins can increase bitterness and astringency (126), the application of processes such as aging-on-lees may help reduce their impact.

The synthesis of vitisins may occur during alcoholic fermentation by the action of yeasts, although their synthesis mostly occurs during wine aging. Vitisins have shown a lower capacity to neutralize free radicals, such as the O_2_^–^ anion, than their precursor anthocyanins (16), although the pyruvic adduct of delphinidin-3-O-glucoside has shown a greater capacity to neutralize OH^–^ and O_2_^–^ anions in comparison to other pyranoanthocyanins, but to a lesser extent than its precursor delphinidin-3-O-glucoside.

During the synthesis of vitisin A, pyruvic acid is incorporated in positions 4 and 5 of the A ring of the precursor anthocyanin, which may reduce its antioxidant capacity. This phenomenon would be related to the loss of the −OH group of carbons 5 and 7 of the anthocyanin, which contributes to its antioxidant activity (168). Thus, greater health benefits would be expected from the consumption of young red wines, where the anthocyanin content is higher.

However, these condensations, according to traditional vinification, are necessary to confer physicochemical and microbiological stability to the wine (152, 156). Although the increase in the content of pyranoanthocyanins does not contribute to increasing the antioxidant capacity concerning the antioxidant capacity conferred by the precursor anthocyanins, the greater stability of the pyranoanthocyanins may contribute to counteracting the loss of antioxidant capacity throughout aging.

#### Aging-on-Lees

One of the red wine aging techniques that has gained importance in recent years is aging-on-lees (AOL), which is characterized by the release of polysaccharides from the cell walls of yeast lees to the wine during aging (169). These polysaccharides can improve, among other attributes, the protection of phenolic compounds against oxidation, allowing, among other benefits, to maintain their antioxidant and anti-inflammatory capacity. In addition, the lees have a higher affinity for oxygen (170), so that in their presence the polyphenols can be protected against oxidation.

Although the content of anthocyanins may decrease during AOL (126, 171) due to adsorption by the lees (126), the application of lees of species such as *S’codes ludwigii* and *S. pombe* (169) may reduce the loss of anthocyanins. However, the lees must come from yeasts with low or no β-glucosidase activity, which can generate hydrolysis, and, therefore, the loss of anthocyanins (146).

Although most studies have focused on the quality of red wine in the aging phase, it has been reported that the addition of polysaccharides before alcoholic fermentation also has a positive effect (Table 5), increasing the content of anthocyanins, phenolic acids, tannins, and antioxidant capacity in the produced red wine (171).

## Current and Future Challenges

The procedures described can be very useful in the winemaking process in order to improve the content and availability of phenolic compounds in the red wine, which once ingested may contribute to improving the health state of the consumer, without neglecting that these compounds cannot replace medicines, since their nature is not curative, but rather as components of the diet that, mainly, can help prevent certain pathologies.

Most of these technologies are mainly aimed at reducing the microbial load in the grape must and improving the physicochemical stability and sensory profile of red wine. However, they can be used to improve the content of bioactive compounds, considering that around 50% of the phenolic compounds are trapped in the red grape residue (pomace) (90) and that not always is used, for example, for food purposes (91).

It is also important to mention that, in most studies, the content of anthocyanins and derivatives, flavanols, and total polyphenols have been evaluated. However, the literature regarding the impact of these technologies on the content of other polyphenols is scarce. For example, in the case of resveratrol, which, according to the existing evidence, is the polyphenol that would provide the most health benefits.

### Industrial Scalability

The scaling up of these technologies is still a pending issue, considering that only high pressures, PEF and US, have been evaluated at the industrial level.

Regarding ultrasound, the OIV, through the Resolution OIV-OENO 616-2019, included its use for the pre-fermentative maceration stage to improve the extraction of phenolic compounds, among other benefits. In the market, it is possible to find systems based on this technology for applications in liquid foods, such as those commercialized by Industrial Sonomechanics (172).

In the case of PEFs, also the OIV (Resolution OIV-OENO 634-2020) has included their use in the pre-fermentative maceration stage, and there is currently industrial equipment for the processing of liquid foods up to 10,000 L h^–1^ and solid foods up to 70,000 kg h^–1^, commercialized by Elea Technology (173). At the winery level, PEF has been applied at intensities between 4.0 and 4.3 kV cm^–1^ on Garnacha musts with workflows up to 2,500 kg h^–1^ (128, 174) obtaining wines with better phenolic contents (Table 3). Regarding PEF intensity, it has applied high intensities between 17 and 30 kV cm^–1^ (Table 3), obtaining improvements in phenolic content and reduction of maceration time (115, 116). However, most of the studies have applied intensities lower than 7 kV cm^–1^ with good results (Table 3), which, in addition, would allow better adaptability at the industrial level, considering that the energy needed to produce high-intensity electric fields determines the characteristics of the PEF generator and, therefore, the cost of the PEF system (118, 130).

Commercially, UHPH systems, such as those offered by Homogenizing Systems, can be found with production capacities up to 1,000 L h^–1^ (175). However, on the company website it is not reported its specific application for winemaking. It is also possible to find the UHPH system patented by the Universidad Autónoma de Barcelona (176) and exploited by Ypsicon Advanced Technologies (177), equipped for winemaking with flow rates up to 50,000 L h^–1^. However, one of the challenges to overcome with this technology is its applicability in red musts containing peels and seeds (grape mash), considering that this technology has been evaluated in liquid foods with particle sizes of less than 0.5 mm (94). An alternative to taking advantage of the benefits of UHPH would be its sequential application in processes that include maceration in the presence of peels and seeds, with subsequent draining, for the later application of UHPH to the pure must, according to the methodology proposed by Vaquero et al. (110). It could also include the application of US, PEF, irradiation, or ozone in the maceration stage, with subsequent draining of the must and application of UHPH to reduce the microbial load and inactivate PPO, protecting polyphenols against oxidation.

Although the technologies described have shown efficacy to improve the extraction of phenolic compounds in the pre-fermentation stage, the decrease in anthocyanin content in wines made from grapes/must treated with HHP and e-beam irradiation is a disadvantage in the small-scale studies, which must be overcome for its industrial applicability, mainly by optimizing winemaking conditions that reduce the decrease in the general content of polyphenols (101, 105, 136).

Although the available literature reports that most of the studies have been carried out with small sample volumes and at the laboratory level, the results obtained have shown the potential of these technologies to improve the phenolic content in red wine, not without highlighting the need for further studies to optimize winemaking conditions with large volumes and in continuous flow systems, for their full implementation in the winery.

Finally, greater dissemination of the benefits of these technologies is necessary, allowing consumers to be aware of their advantages in the production of red wines, such as the reduction of the necessary doses of SO_2_, which has been related to different health problems in the consumer (95).

## Conclusion

There is ample evidence regarding the benefits of wine on health, especially red wine, due to its higher content of phenolic compounds, which can help maintain good health and prevent diseases, being moderate consumption vitally important. The first part of the review summarizes some studies conducted, mostly with information available on the preclinical level, which contributes to a better understanding of the mechanisms by which phenolic compounds could act in the human body (clinical level), taking advantage of their antioxidant, anti-inflammatory, antitumor, antithrombotic, antiatherogenic, and antimicrobial activity, among others. According to the bibliography consulted, in the last years various studies address specific procedures aimed at improving the bioactive profile of red wine. In this sense, the second part of the review describes technological strategies that can contribute to increasing or at least maintaining, the content of polyphenols in red wine. In the pre-fermentation stage, extraction can be increased by treating the grape berries with high pressures, PEF, US, e-beam radiation, and ozone, while, at the fermentative level, yeasts with low adsorption of polyphenols, low anthocyanin-β-glucosidase (anthocyanase) activity, and high production of pyranoanthocyanins and/or their precursor molecules could be very useful. During aging, although in most cases the content of polyphenols can be reduced, AOL can maintain the levels of these compounds in the wine, in addition to chemical processes that modify its structure, such as the synthesis of pyranoanthocyanins, polymerization of anthocyanins and flavanols, anthocyanin-tannin condensation, among others, maintaining to a certain extent the bioactive profile of red wine, and, therefore, transmitting its health benefits to the consumer.

## Author Contributions

RV conceptualized, drafted, and revised the manuscript. ML-C drafted the manuscript. Both authors reviewed and approved the finalized version of the manuscript.

## Conflict of Interest

The authors declare that the research was conducted in the absence of any commercial or financial relationships that could be construed as a potential conflict of interest. The handling editor AM declared a past collaboration with the author RV.

## Publisher’s Note

All claims expressed in this article are solely those of the authors and do not necessarily represent those of their affiliated organizations, or those of the publisher, the editors and the reviewers. Any product that may be evaluated in this article, or claim that may be made by its manufacturer, is not guaranteed or endorsed by the publisher.
